# Extensive Exophytic Gum Swelling: A Case Study

**DOI:** 10.3390/reports8020052

**Published:** 2025-04-21

**Authors:** Abdullah Saeidi, Mahir Mirah, Albraa Alolayan, Hattan Zaki, Shadia Elsayed

**Affiliations:** 1 Department of Oral and Maxillofacial Diagnostic Sciences, College of Dentistry, Taibah University, Madinah 42353, Saudi Arabia; abolayan@taibahu.edu.sa (A.A.); hzaki@taibahu.edu.sa (H.Z.); ssayed@taibahu.edu.sa (S.E.); 2 Department of Restorative Dental Sciences, College of Dentistry, Taibah University, Madinah 42353, Saudi Arabia; mahirmirah@gmail.com; 3 Health and Life Research Center, Taibah University, Madinah 42353, Saudi Arabia

**Keywords:** pyogenic, granuloma, dental lesion, extensive, case study

## Abstract

**Background and Clinical Significance:** Large extensive intraoral dental swelling is uncommon in adults, and we report a rare case of large exophytic oral granulomatous tissue. A complete explanation of diagnostic steps and surgical treatments is addressed, as well as a thorough review of the literature, and a discussion of this interesting case is provided to underline the need for recognition of these vascular pyogenic proliferative lesions and discuss proper management based on the underlying cause. **Case Presentation:** A 21-year-old Afghan female patient who presented with an extensive intraoral pale red, friable lesion that bled easily. It was 15 × 15 mm in size related to the lower second molar, and interfered with occlusion and extended to cover the buccal and lingual surfaces of adjacent teeth. **Conclusions:** The presentation of this rare large reactive vascular proliferative condition of oral posterior gingiva, contribute to a better understanding and the growing body of evidence on the PG. The findings emphasize the importance of early intervention tailored to the patient’s age, lesion location, and underlying causes and patient education to prevent extensive dental tissue destruction.

## 1. Introduction and Clinical Significance

Pyogenic granuloma (PG), also known as lobular capillary hemangioma, is a common benign inflammatory reactive hyperplastic dental lesion of the oral gingival tissue [1,2]. While typically small, cases of unusually large PGs have been reported, with some exceeding 5 cm [1,3]. These vascular lesions can cause difficulties in swallowing, chewing and breathing. Clinically, PGs usually present as smooth, lobulated exophytic masses, ranging from pink to reddish-purple in color, and are often red and hemorrhagic and may bleed easily [4]. PGs present typically on the gingiva but could occur occasionally on other oral sites [5].

They can occur at any age, but are most commonly seen in particular in young adults during their second decade of life [6]. They have no gender predilection, though some studies suggest a slight female predominance due to hormonal influences. When it occurs in pregnant women, it is usually called pregnancy-related PG and is called “granuloma gravidarum” or pregnancy tumor [7]. Although it is reported to be associated with hormonal changes during pregnancy, particularly estrogen, large PGs have been observed in both young males and postmenopausal females, due to local irritation or trauma, and this is considered a challenging etiological factor [1,6].

The exact cause of pyogenic granuloma is unknown, but several factors are associated with its development: chronic dental traumatic irritation or infections may play a role in some cases, and oral contraceptive use or hormonal fluctuations can trigger PG. Furthermore, vascular endothelial growth factor (VEGF) overexpression may contribute to the rapid growth of these lesions. In addition, certain drugs, such as retinoids, antiretroviral therapy, and targeted cancer therapies, have been linked to PG development.

Pyogenic granuloma is typically managed based on its size, location, and symptoms. Treatment typically involves the standard scalpel surgical excision treatment, with rare recurrence [8]. This allows for histopathological confirmation of the diagnosis. In some cases, electrocautery is used to remove the lesion and control bleeding. Freezing cryotherapy, freezing the lesion with liquid nitrogen, is effective for smaller lesions. CO2 or pulsed dye lasers could also be used [2]. Recurrence rates are relatively high (up to 15–20%), especially if the lesion is not fully excised, and so proper wound care and follow-up are essential.

Therefore, the aim of the present study was to report an interesting case of extensive chronic pyogenic exophytic mass, highlighting its clinical, radiographic, and histopathological features, and to review the current literature on its etiology, pathogenesis, and management.

## 2. Detailed Case Presentation

### 2.1. Case Presentation

In January 2025, a 21-year-old Afghan woman presented at Taibah University College of Dentistry’s endodontic clinic with complaints of pain and intraoral swelling associated with a big mass related to her lower second left molar. The swelling had begun a year ago. The prior medical history of the patient was normal. Dental history showed generalized periodontal gingivitis and carious lower first and second molars, and the main complaint of the patient was the intraoral enlargement of the lower second molar region, which was odorous, irritating, and caused bleeding during chewing and eating. At first, the discomfort was moderate, but it gradually increased.

### 2.2. Clinical Examination

**Figure 1:**
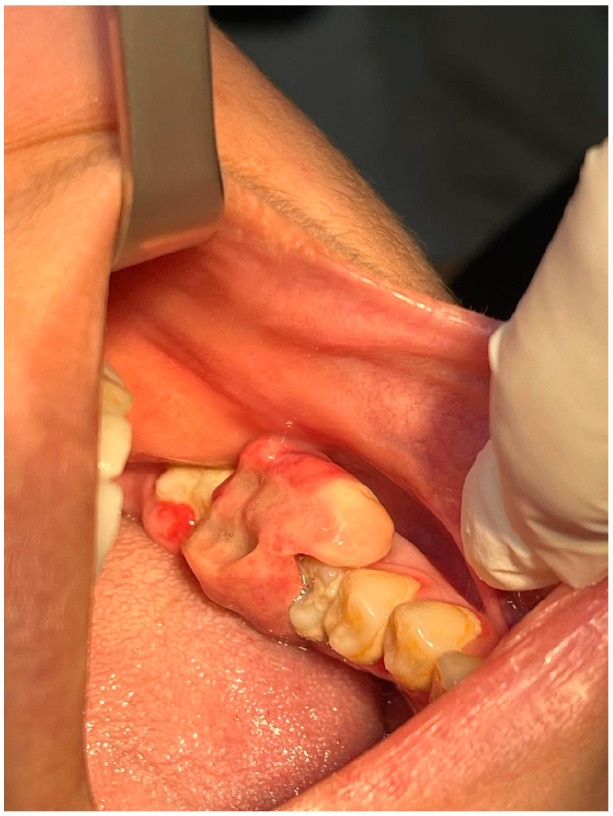
Intraoral photograph showing extensive exophytic large soft tissue mass covering the occlusal surface of the lower first and second left molars.

An intraoral examination showed a large, pale red soft tissue lesion covering the occlusal surface of the left lower first and second molar teeth. The lesion was a big, pedunculated mass and covered both molar teeth’s buccal and lingual surfaces. The lesion was firm but movable, and it felt slightly painful to the touch. It bled when moved, which made it uncomfortable for the patient and made it harder for them to chew and eat. Although the surrounding gingiva seemed irritated, there were no obvious signs of periodontal infection. All of the patient’s vital signs were within normal limits, and there were no indications of an infection or elevated body temperature. The patient had poor oral hygiene in general, and she had not brushed the left side of her jaw in months (Figure 1).

### 2.3. Radiographic Examination

**Figure 2:**
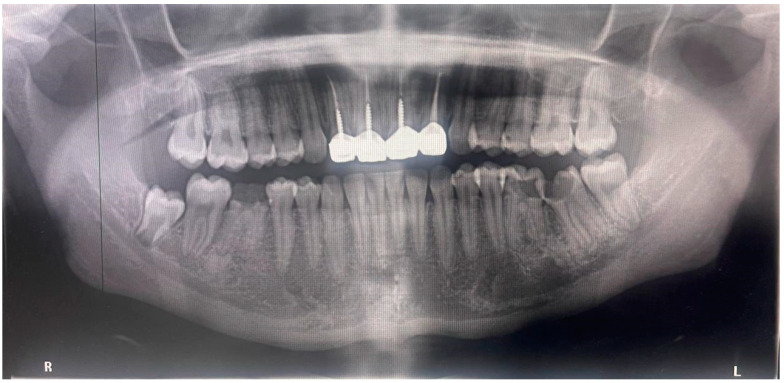
Orthopantomogram showing badly decayed lower left first and second molar.

An extraoral orthopantomogram revealed extensive caries in the lower left first and second molar teeth that extended to the pulp chamber and caused loss of the coronal tooth structure. There was also an absence of periapical disease at the apical region of the second molar (chronic hyperplasia without necrosis). There was no indication of periapical radiolucency. The root structure remained intact, with no external resorption or considerable alveolar bone loss seen. The carious right first mandibular molar showed periapical hypercementosis, several endodontic and restored anterior maxillary teeth, and an impacted lower right wisdom tooth (Figure 2).

### 2.4. Surgical Management

The patient was informed about the possible causes of the lesion and its development before undergoing surgical excision. Treatment options included root canal treatment because of pulp necrosis and because irreversible damage was evident, or surgical extraction. The patient agreed to the proposed plan and provided signed informed consent before surgery. The presentation of the present case was reviewed and accepted by Taibah Dental College and Hospital’s ethical committee, with the approval number (TUCDREC/080225/ASaeidi).

**Figure 3:**
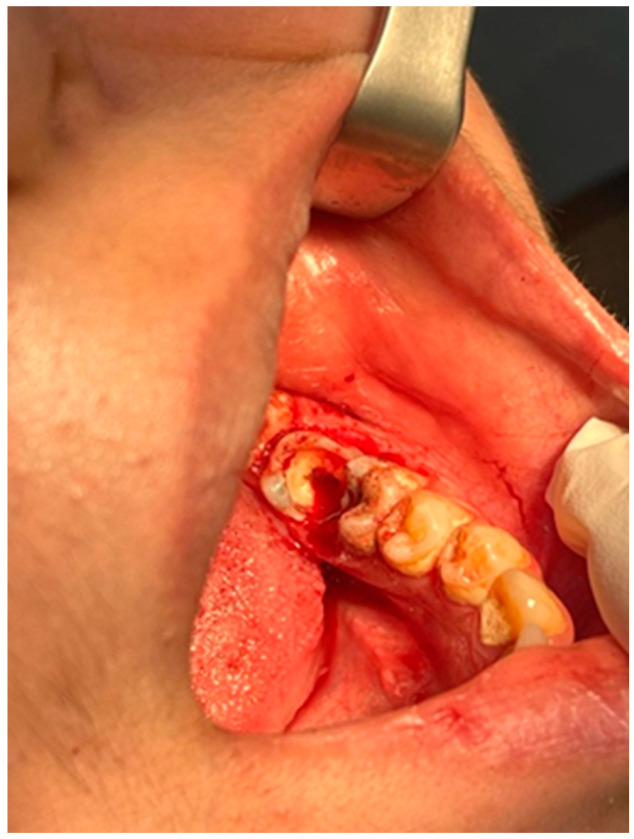
Intraoral photograph showing complete surgical excision of the lesion and controlled bleeding.

Local anesthesia was administered using a 4% articaine via inferior alveolar nerve block and long buccal infiltration. The lesion was dissected and lifted from its base with a bone curette, and a deep excision from its pedicle inside the tooth carious cavity was performed, while gauze pressure kept the surgical fields in hemostasis and bleeding under control until the entire lesion was eliminated, followed by full caries eradication and periodontal curettage. A temporary filling was inserted, and another appointment for endodontic treatment was made. The lesion size was 15 × 15 mm. The patient was instructed to practice thorough and rigorous oral hygiene, including using chlorohexidine mouthwash 5 times per day and cleaning the teeth twice each day (Figure 3).

### 2.5. Results

Based on clinical and radiographic findings, an extensive large chronic hyperplastic granulomatous lesion was confirmed and should be differentiated clinically from peripheral fibroma, gingival hyperplasia, pyogenic granuloma, or peripheral giant cell granuloma, polyp, lobular capillary haemangioma, and squamous cell carcinoma (Table 1).

**Figure 4:**
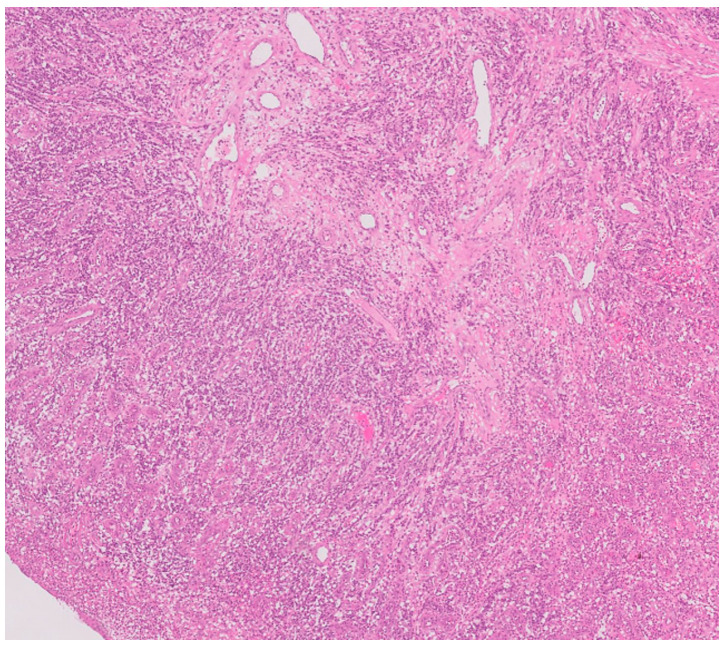
Microscopic histopathological examination revealed a large area of ulceration covered by fibrin mesh intermixed with subacute inflammatory cell infiltrate (10×). The underlying connective tissue shows proliferation of small blood vessels in a lobular fashion. Areas of hemorrhage area are seen.

Histological microscopic examination of the excisional biopsy of the lesion revealed a specimen surfaced by para-keratinized stratified squamous epithelium characterized by a large area of ulceration covered by fibrin mesh intermixed with subacute inflammatory cell infiltrate. The underlying connective tissue shows proliferation of small blood vessels in a lobular fashion. Areas of hemorrhage area can be seen. No evidence of dysplasia or malignancy was observed (Figure 4).

A follow-up visit after one week was scheduled and the healing of the lesion was excellent. Postoperative recovery was uneventful, with no recurrence noted during the follow-up period. The patient reported a significant improvement in comfort and function and expressed satisfaction with the outcome, highlighting relief from previous symptoms and improved outcomes.

## 3. Discussion

Extensive PG as a benign vascular gingival swelling is rare in adults, and we present here a large extensive intraoral pyogenic lesion in a 21-year-old Afghan national female patient who had large intraoral dental swelling related to a lower second molar, interfering with occlusion and extending to cover the buccal and lingual surfaces of adjacent teeth. The present case findings align with previously reported cases of chronic hyperplastic pyogenic granuloma, which could affect individuals of all ages, with peak incidence in the second and fifth decades of life and a higher prevalence among women due to hormonal influences. The gingiva is the most common site [9,10], and its characteristic clinical features and favorable prognosis with timely management should be emphasized, as well as the importance of correlating clinical characteristics with diagnostic investigations [11,12].

Pyogenic granuloma is common in Saudi Arabia. In a retrospective study reporting analysis of 624 gingival and alveolar ridge overgrowths, PG accounted for 38% of cases as the most prevalent reactive hyperplastic lesion [10]. Additionally, according to Wayli et al., 2016 [13] in another study in Riyadh, it is considered among the most common oral mucosal lesion in Saudi females. However, in southwestern Saudi Arabia a lower prevalence was reported of only 7% of biopsied oral and maxillofacial lesions [14]. This prevalence discrepancy may be attributed to differences in study design and population. On the other hand, in an Iranian population study, the gingiva was the most affected site (70.3%), followed by the tongue, lips, and buccal mucosa [15].

PG etiology includes local irritation, trauma, and poor oral hygiene, which is a significant contributing factor in the present case. According to the literature demonstrating the link between pregnancy and unfavorable periodontal diseases outcomes due to hormonal changes and high levels of estrogen, many pregnant women lack adequate awareness about the importance of maintaining oral health during pregnancy [16,17]. Despite the fact that tobacco smoking may also be a contributing factor to the onset and persistence of reactive oral lesions, no research has identified a link between the formation of pyogenic granulomas and tobacco-related oral lesions [18], and the present case did not use tobacco.

The present histopathology aids in differentiating PG from related lesions that have different vascular patterns, cellular compositions, and the presence of mineralized tissue, such as peripheral giant cell granuloma, peripheral ossifying fibroma, and hemangioma. Clinically, color was reported as a distinguishing feature for these entities and so differentiating pyogenic granuloma (PG) from other vascular proliferative lesions is essential for accurate diagnosis and appropriate management [19]. PG is characterized by lobular capillary proliferation, with a noticeable granulation tissue-like appearance, as our histographic evaluation showed [20].

In the present case, the female patient’s pain threshold appeared to be very high, and adaption of the opposing molar on the lesion’s superior surface is clearly visible, indicating a chronic condition and the remarkable adaptability of intraoral connective tissue cells. These findings highlighted that the lesion’s low pain characteristics, despite its large size, can lead to delayed treatment, which emphasizes the need for early intervention in preventing significant dental tissue damage [21]. Studies have also shown that the strong vascular supply and immune response of young people contribute to the hyperplastic response, as seen in this case. Granulomatous vascular growth includes stem cells and immune cells that combine to prevent infections, trigger immunological responses, and maintain tissue homeostasis. In cases of dental traumatic irritation, the fight between neurovascular ingrowth and bacterial invasion dictates infection outcomes, with an intact neurovascular supply allowing for efficient immune defenses [22,23].

In the present case, management was aimed at keeping the damaged tooth as a functional unit of dentition, especially in adults, when extraction may cause a variety of dental problems [24]. The excision of the lesion, followed by endodontic therapy, aligns with management strategies discussed by previous cases that have demonstrated that prompt surgical intervention, combined with restorative procedures, ensures favorable outcomes and prevents progression to apical periodontitis or systemic complications. Conservative treatment approaches have shown satisfactory results in maintaining afflicted teeth as functional dentition units [25].

Clinically, PG can present as a sessile lesion or raised mass as presented here, with bleeding being a common complaint. While PGs have no malignant potential, they may be clinically confused with malignancies due to their appearance and pattern of growth [4]. Interestingly, pyogenic granuloma exhibits extraordinary resistance to oncogenesis. This resistance contrasts with higher malignant potential in other tissues, such as the gut and brain [26]. More research is needed to better understand the distinct features of dental pyogenic cells that protect against malignant transformation.

Treatment of gingival PG typically involves traditional surgical excision and removal of irritants, but this may not prevent recurrence, with a low recurrence rate except for in pregnancy-related cases, where spontaneous resolution may occur [27,28]. In contrast to other reports, it has a high tendency to recur after excision [29]. The recurrence rate could be as high as 15.8% [30]. To minimize recurrence, complete excision down to the periosteum, as performed in the current case, and removal of irritants are recommended. Adjunctive approaches have been recommended, including a nonsurgical protocol involving strict oral hygiene, scaling, and root planning and the use of platelet-rich fibrin membrane to cover the exposed surface after excision [28,30]. Meanwhile, in skin PGs, other approaches could be more useful, such as beta-blocker Topical timolol gel, which can be effective for small PG lesions, particularly in children. Topical steroids may help reduce inflammation and size in some cases. Sclerotherapy via the injection of sclerosing agents can be used for highly vascular lesions. In pregnancy-related PG, lesions’ hormonal changes may contribute to the development of gigantic lesions, which often regress spontaneously after delivery, so conservative management is preferred unless symptomatic [31]. Interestingly, these lesions can persist even after pregnancy, as reported in another case, where a pyogenic granuloma remained for two years post-delivery [7]. Accurate diagnosis through histopathological examination is crucial, as PG can be misdiagnosed as other gingival lesions.

The current study is a single case report and therefore does not allow for statistical analysis or wide epidemiological conclusions. Despite this limitation, this case study provides a useful comprehensive, patient-centered investigation of a pyogenic granuloma lesion.

## 4. Conclusions

The presentation of this rare large reactive vascular proliferative condition of oral posterior gingiva contributes to a better understanding and to the growing body of evidence on PGs. The findings emphasize the importance of early intervention tailored to the patient’s age, lesion location, and underlying causes and patient education to prevent extensive dental tissue destruction.

## Figures and Tables

**Table 1:** Differential diagnosis of oral pyogenic granuloma lesions.

Lesion	Size	Consistency	Shape	Surface Color	Bleeding	Relation to Tissues
Pyogenic Granuloma (PG)	0.5–2.5 cm (can vary)	Exophytic Soft to firm	Sessile or pedunculated	Reddish Smooth or lobulated	Bleeds easily	Usually on gum, attached to soft tissue
Peripheral Giant Cell Granuloma (PGCG)	Up to 5 cm	Firm, rubbery	Usually sessile	purplish Smooth, sometimes ulcerated	May bleed	Exclusively on gingiva or alveolar ridge
Peripheral Ossifying Fibroma (POF)	<2 cm	Firm	Sessile	Pinkish smooth May show calcification histologically	Minimal bleeding	Gingiva (interdental papilla)
Irritation gingival hyperplasia	<1.5 cm	Firm	Sessile	pale Smooth, normal mucosal color	Rarely bleeds	chronic trauma mucosal surface, mobile
Lobular Capillary Hemangioma (LCH)	Variable, often <2 cm	Soft, compressible	Sessile or pedunculated	Red–blue, translucent	Vascular Bleeds easily	Submucosal, may blanch with pressure
Squamous Papilloma	<1 cm	Soft	HPV-related, pedunculated	White–pink, wart-like papillary or cauliflower-like	Rarely bleeds	Attached to mucosal surface
Metastatic Tumor (rare)	Variable	Firm	Sessile or irregular	Ulcerated, nodular	May bleed	Malignant, fixed to underlying structures

## Data Availability

All data are available on request from the corresponding author.
